# Dual Processes of Oculomotor Capture by Abrupt Onset: Rapid Involuntary Capture and Sluggish Voluntary Prioritization

**DOI:** 10.1371/journal.pone.0080678

**Published:** 2013-11-19

**Authors:** Feng Du, Yue Qi, Xingshan Li, Kan Zhang

**Affiliations:** 1 Key Laboratory of Behavioral Science, Institute of Psychology, Chinese Academy of Sciences, Beijing, China; 2 University of Chinese Academy of Sciences, Beijing, China; University of Muenster, Germany

## Abstract

The present study showed that there are two distinctive processes underlying oculomotor capture by abrupt onset. When a visual mask between the cue and the target eliminates the unique luminance transient of an onset, the onset still attracts attention in a top-down fashion. This memory-based prioritization of onset is voluntarily controlled by the knowledge of target location. But when there is no visual mask between the cue and the target, the onset captures attention mainly in a bottom-up manner. This transient-driven capture of onset is involuntary because it occurs even when the onset is completely irrelevant to the target location. In addition, the present study demonstrated distinctive temporal characteristics for these two processes. The involuntary capture driven by luminance transients is rapid and brief, whereas the memory-based voluntary prioritization of onset is more sluggish and long-lived.

## Introduction

Decades of psychological research have shown that some salient events, such as the abrupt onset of a new object [1], [2], the sudden disappearance of an existing object [3], the presence of an irrelevant feature singleton [4], and the onset of motion [5], [6], can capture attention in a stimulus-driven fashion. The stimulus-driven capture of attention refers to attentional selection that is irrelevant to or even against people's intention. The hallmarks of stimulus-driven capture are involuntary, rapid and short-lived [7]. Attentional capture by an abrupt onset is perhaps the prototypical example of stimulus-driven capture [7], [8]. In fact, an abrupt onset captures not only covert attention but also eye gaze. For example, when observers were required to make a saccade to a color-singleton target, the abrupt onset of an irrelevant distractor disrupted the saccade toward the singleton target. Instead, observers tend to make eye movements toward the new distractor [9], [10]. In addition, the onset has a similar disruptive effect on contrast discrimination [11]. Some recent studies have also shown that the onset is more effective in capturing gaze than is color change [12], [13].

However, there is disagreement about what actually causes onset capture. The luminance transient theory suggests that the onset capture is solely due to the unique luminance transient associated with the onset. As a result, onset capture can be eliminated by a visual mask in between cues and targets [14], [15]. In contrast, the new object theory proposes that a new object can capture attention even without a unique luminance transient associated with onset [16], [17]. Thus it is the new perceptual object that captures attention involuntarily [18]–[20]. Researchers also debate whether onset capture is under voluntary control. Many early studies suggested that onset capture is involuntary because it occurs even when onsets are irrelevant to the task [1]–[4]. Since then, ample studies have shown that onset capture can be modulated by the particular goal of observers. For example, Folk and colleagues found that onsets of uninformative pre-cues captured attention if and only if targets also appeared abruptly [21], [22]. Conversely, onsets often fail to capture attention if they do not match the features that define the target [23]–[28]. In addition, onsets can be ignored when observers have sufficient time to fixate the target location prior to the appearance of new objects [2], [3], [29]. This location based top-down control can be applied to not only the covert orienting of attention but also the overt orienting of eyes: oculomotor capture by onset does not occur if target locations are pre-cued [9], [10].

To some extent, the two competing theories were reconciled by a series of studies on oculomotor capture in real-world scenes [13], [30]–[32]. Brockmole and colleagues asked participants to either memorize or view real world scenes and presented a new object abruptly in each scene. The new object appeared either when participants were fixating or when they were making a saccade. The authors reported that the new object was fixated more often than chance whether it appeared during fixations or saccades [13], [30]–[32]. Since the luminance transient can be largely suppressed during saccades [33], these results confirmed that the appearance of a new object yields attentional prioritization even in the absence of a transient signal. However, they reported that the new object was less likely to induce oculomotor capture when it appeared during saccades relative to during fixations. These studies showed that the new object that appeared during saccades was fixated slower and less often than those coincident with fixations. Thus a luminance transient did play an important role in guiding gaze though it is not the sole cause of onset capture [30]–[32].

Based on these results, Brockmole and colleagues proposed that oculomotor capture of onset is not solely driven by the luminance transient. When onsets occur during fixations (the global luminance transients associated with onsets are intact), the transient-driven capture is a rapid and involuntary process [30], [31]. However, when onsets occur during saccades (the luminance transients associated with onsets are suppressed in this case), the prioritization of onset is a slower process which is mainly guided by the contents in working memory. As a result, the prioritization of onset during saccades is sensitive to the viewing time of a scene: reducing scene viewing time prior to the onset eliminated prioritization, whereas longer viewing time of the scenes increased prioritization [31]. In addition, the identity consistency between new objects and scenes also influenced the prioritization of new objects during saccades, with new inconsistent objects fixated sooner than new consistent objects. These results suggested that the memory-based prioritization is under top-down control, or at least is “partially controlled by object identity and meaning” [32].

The present study aims to examine the dual processes model by tracking eye movements in a classic visual search task. First, a visual mask is inserted between the cue and the target to disrupt the unique luminance transient associated with onset [14], [16]. According to the luminance transient theory of onset capture, visual masks eliminate the unique luminance transient of onset, thus completely suppressing oculomotor capture of onset [14], [15]. However, the dual processes theory would expect that an onset still has attentional priority even with the disruption of a visual mask. Since visual masks are only expected to disrupt the transient-driven capture of attention and leave the memory-based prioritization intact, visual masks can only reduce but not completely wipe out the onset advantage. Yet, most of the fore-mentioned studies used RT facilitation as a measure of onset capture. Previous studies showed that attentional capture could be directly reflected by eye gaze [9], [10]. Actually eye movements provide a more straightforward and fine-grained measurement of the steps of processing leading up to the responses, whereas RTs only provide an aggregate measurement of these processes. By tracking eye movements, the present study is the first to examine whether a visual mask can completely wipe out the oculomotor capture by onset.

Secondly, Brockmole and colleagues suggested that memory-based prioritization is a voluntary process because it is influenced by the contents in working memory [32]. However, it is still unknown whether the memory-based prioritization of new objects is truly controlled by knowledge of the target location. It is also a mystery whether the transient-driven capture of onset is truly involuntary in the sense that the process occurs even when onset is never a target. We manipulated the probability with which an onset can be a target in two experiments here to address these two questions. Since the new object was never a target in Experiment 2, the probability of fixating on the new object without a mask should still exceed chance level after onset, mainly reflecting an involuntary capture of attention. But if the memory-based prioritization of onset is under top-down control, one would expect that the probability of fixating on the new object with the presence of a mask should be as low as chance level.

Finally, the present study used a visual search task which is different from the tasks in the Brockmole et al studies [30], [31]. The prolonged prioritization of new objects during both fixations and saccades in their studies were usually not reported in previous studies of oculomotor capture [9], [10]. It is unknown whether observers still have prolonged prioritization of new objects when they are required to perform a speeded visual search task. With time pressure, the present experiment served as a more stringent test of the temporal characteristics of dual processes underlying onset capture.

## Experiment 1

This experiment used a visual search task to examine whether a visual mask between cue and target can completely remove onset capture. The luminance transient theory predicts absence of onset capture because a visual mask can eliminate the unique luminance transient associated with an onset. However, the dual processes theory suggests that onset capture relies on not only the transient-driven capture but also the memory-based prioritization of a new object. Thus it predicts that an onset still has attentional priority even when a visual mask disrupts the unique luminance transient associated with an onset. In addition, previous studies found that the memory-based prioritization of a new object is a long-lasting process [30], [31]. However, this long-lasting prioritization of a new object might be specific to the tasks in those studies. With time pressure, the present experiment served as a more stringent test of the temporal characteristics of the dual processes underlying onset capture. If there is a prolonged effect of onset under the influence of visual masks, the new object should capture attention at the second ordinal fixation or even at the third ordinal fixation with visual masks, whereas onset capture only occurs at the first ordinal fixation with no mask.

### Methods

#### Participants

This study was approved by the internal review board of the Institute of Psychology, Chinese Academy of Sciences. Twelve undergraduate and graduate students (6 males, 24.17±1.95 yrs) gave informed consent before they participated in this experiment. All of them had normal or corrected-to-normal vision. Participants were naïve with regard to the purpose of the experiment. Each participant received cash compensation for their participation.

#### Procedure and Apparatus

Eye movements were recorded by an Eye-link 1000 eye tracker which sampled monocularly at 1000 Hz (SR Research). Stimuli were presented on a 19-inch CRT monitor at a resolution of 1024×768 pixels with a refresh rate of 60 Hz. A chin-rest was located 60 cm away from the monitor to minimize head movements. Viewed from this distance, the screen is 28° in width and 21° in height. The participants responded by pressing buttons on a Microsoft SideWinder gamepad. The eye tracker was calibrated at the beginning of the experiment and the calibration was validated as needed. For calibration and validation, subjects looked at a dot that was presented at each of nine locations of 3 by 3 grids in a random order (the maximum error permitted for validation throughout the experiment was 0.5° of visual angle).

**Figure 1:**
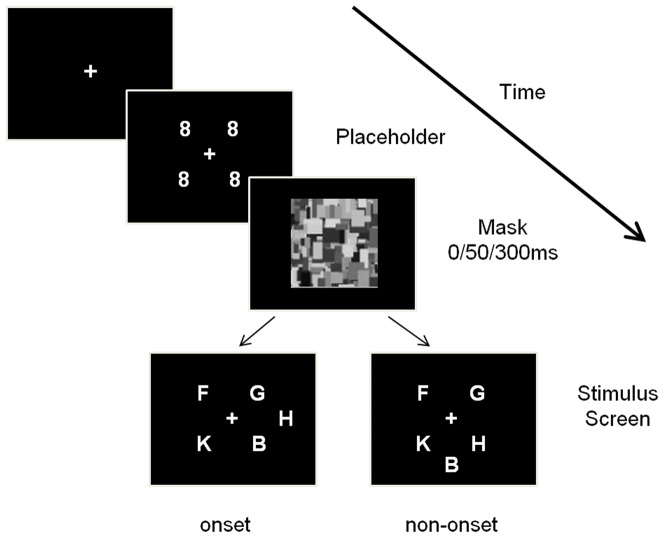
The sequence of events of two trials in Experiment 1. The target letter “H” or “S” can be either an onset target or a non-onset target.

The sequence of events of each trial is illustrated in Figure 1. Drift correction was conducted at the beginning of each trial. For drift correction, a fixation circle was present at the centre of the display and participants had to press a button while they fixated on the circle. A central fixation cross appeared after drift correction, and the participant had to keep fixating at the central cross (within an imaginary circle centered on the cross with a radius of 2°) for 800 ms to continue with the trial. After that, four placeholders were presented at four evenly-spaced locations (45°, 135°, 225° and 315°clockwise from 12 o’clock respectively) on a virtual circle with a radius of 5° centered on the fixation cross. Each placeholder was a white figure eight, 0.4° in width and 0.7° in height. Participants were required to keep fixating at the cross for another 1000 ms. For one third of trials, there was no mask between the four placeholders and a search array of five letters. For the rest of the trials, the four placeholders were replaced by a random visual mask of mosaic with a size of 18° in width and 16° in height which appeared for either 50 ms or 300 ms with equal probability. Then the search array of five letters was presented immediately after the mask. The placeholders would not re-onset after the visual mask. The four letters that appeared at the same locations as the four placeholders were referred to as old objects. The fifth letter was presented at one of four evenly-spaced locations (0°, 90°, 180°, 270° clockwise from 12 o’clock) on the same virtual circle as that of placeholders. Therefore, this fifth letter was a new object. The target letter was randomly chosen from the letters “H” or “S” and presented at one of five locations with equal probability. Thus the target could appear as an onset letter for 20% of the total trials. The four remaining letters were distractors and randomly chosen from B, E, F, N, P, R, U, and X, without repetition in every trial. Participants were instructed to press the left button when they saw the letter “H” and click the right button when they saw “S”. They were required to make accurate responses as quickly as possible.

Participants received four blocks of trials which lasted about half an hour. The first block consisted of 32 trials for practice, and each of the other three blocks consisted of 80 trials, for a total of 240 trials for the experiment. Each trial was randomly chosen.

#### Design

The experiment had two within-subject variables: (1) the target onset condition. The target could be either a non-onset letter or an onset letter, producing the *non-onset target* condition and the *onset target* condition; (2) the mask condition (no mask, presence of a 50 ms mask, or presence of a 300 ms mask). Sixteen trials were assigned to each of the three mask conditions when the target was an onset letter, producing forty-eight trials for the *onset target* condition. Sixty-four trials were assigned to each of the three mask conditions when the target was a non-onset letter (the *non-onset target* condition). As a result, the target appeared as an onset letter for only 20% trials, which was designed to prevent participants from paying special attention to onsets.

#### Eye movement Data analysis

The eye movement data were analyzed using Eyelink Data Viewer software. A saccade was defined as an eye movement with a velocity greater than 30 deg/s and an acceleration greater than 8000 deg/s^2^. Fixations were defined as periods of relatively stable gaze between two saccades; however, 658 fixations that were shorter than 80 ms (approximately 5.0%) were excluded from the analyses. The whole area of interest (AOI) was an imaginary ring centered on the central fixation which had an inner radius of 1° and an outer radius of 9°. This ring-shaped AOI was evenly divided into eight sub-AOIs, each of which was centered on one of eight possible letter locations. Any fixations into the eight sub-AOIs were considered as valid fixations on letters in the search display.

### Behavioral Results

**Figure 2:**
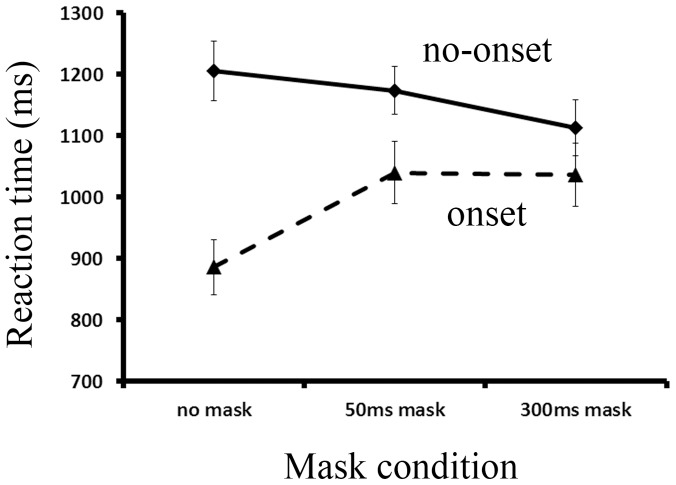
The mean reaction time of all conditions in Experiment 1. The error bar is one standard error.

The mean RTs of correct responses in Experiment 1 are illustrated as a function of the target onset condition and the mask condition in Figure 2. A 2 (target onset condition) ×3 (mask condition) ANOVA revealed a significant effect of the target onset, F (1, 11)  = 34.339, *p*<.001, *η_p_^2^* = *0.757*, with faster responses to an onset target than a non-onset target. This confirmed the presence of onset capture. There was also a significant effect of mask condition, F (2, 22) = 7.109, *p* = .004, *η_p_^2^* = *0.393*. Most importantly, the interaction between target location and mask duration was significant, F (2, 22) = 18.531, *p*<.001, *η_p_^2^* = *0.628*, with the smallest onset capture effect in the 300 ms mask condition and the largest onset capture in the no mask condition. However, further multiple comparisons with Bonferroni correction showed that participants responded faster to an onset target than a non-onset target across all three mask conditions (all *p*<.05). These results indicated that a visual mask did reduce onset capture by disrupting the unique luminance transient associated with onset, but even with the presence of a visual mask, the onset targets still had priority over non-onset targets.

The mean accuracy of Experiment 1 is listed in Table 1. The average accuracies in all conditions were above 98%. The ANOVA test revealed no effects of target location F (1, 11) = 2.80, *p* = .122, *η_p_^2^* = *0.203*, nor effect of mask duration F (2, 22)< 1. The interaction was also not significant, F (2, 22)< 1.

**Table 1:** The mean accuracy of all conditions in Experiment 1.

	No mask	50 ms mask	300 ms mask
**No-onset**	98.57±.36	98.31±.78	98.31±.45
**Onset**	98.96±.70	99.48±.52	99.48±.52

### Eye Movement Results

Analysis of eye movements focused on the probability with which participants fixate on the new object region of the scene. Four ordinal fixations following the appearance of the search display (denoted Fixations 1, 2, 3 and 4) were examined. For example, Fixation 1 denotes the first fixation that participants selected after the new object appeared. To establish a chance level of fixating on a non-onset letter, we used the probability that participants fixated on a non-onset and non-target letter. We refer to this as the *baseline viewing rate*. If a new object captures attention, one would expect that the probability of fixating the new object should exceed this baseline viewing rate right after its appearance [16].

On average, the new object was fixated more often when it was the target (in the onset target condition, *M* = 92.7% of the first four ordinal fixations following onset) than when it was not (in the non-onset target condition, *M* = 55.1% of the first nine fixations), *t*(11) = 11.616, *p*<.001. Since onset capture is best demonstrated in the non-onset target condition, we analyzed the non-onset target condition and the onset target condition separately.

#### Oculomotor capture in the non-onset target condition

**Figure 3:**
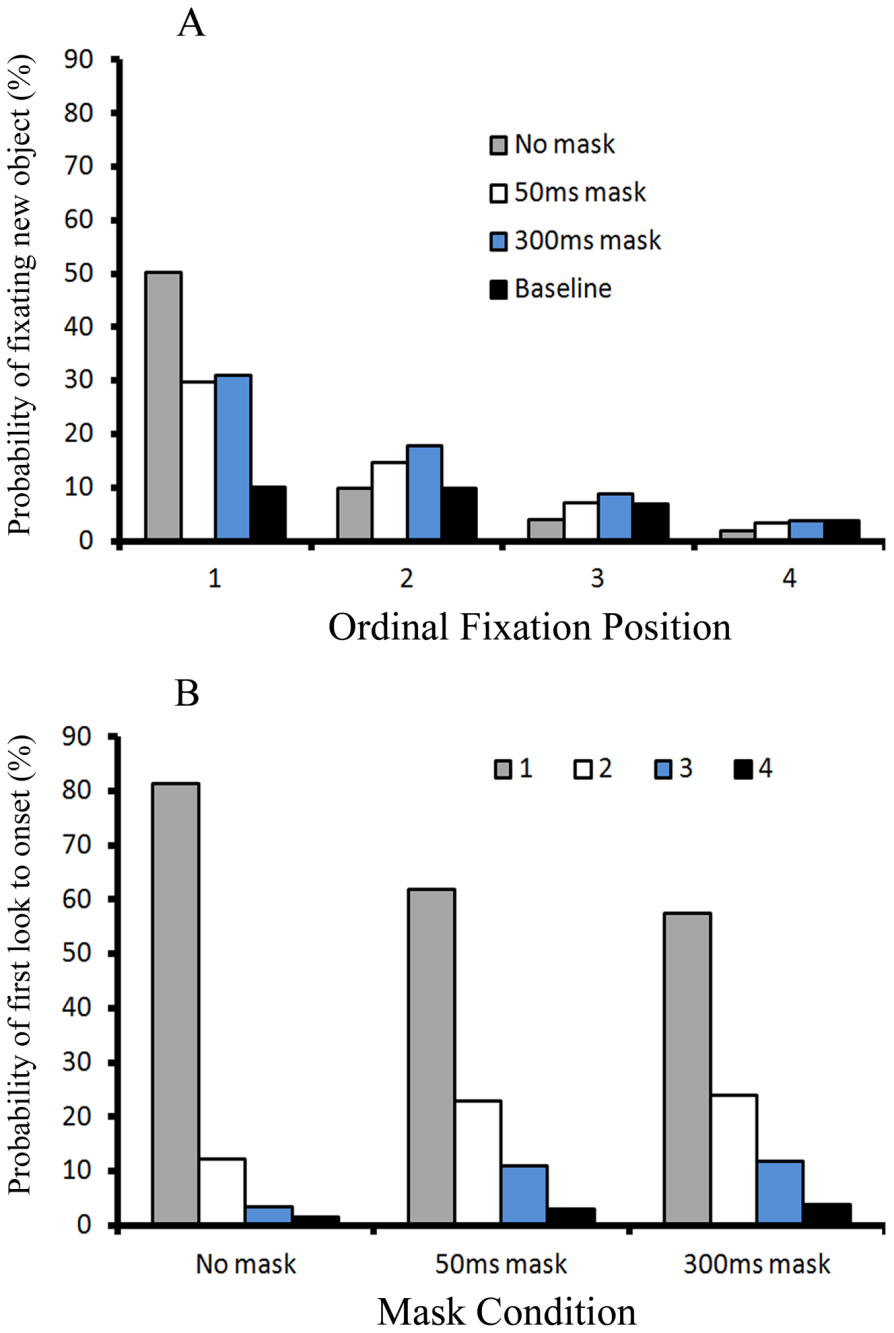
Fixation results in the non-onset target condition of Experiment 1. Panel A illustrates the probability of fixating new objects under the non-onset target condition in Experiment 1; Panel B illustrates the probability of first look to onset for the three mask types under the non-onset target condition in Experiment 1.

When the new object was not a target (the non-onset target condition), the probability of fixating new objects is illustrated as a function of the ordinal fixation position and the mask condition in Figure 3A. A 4 (ordinal fixation position) X 4 (three mask conditions and the baseline viewing rate) ANOVA revealed a main effect of the ordinal fixation position, *F*(3, 33) = 121.844, *p*<.001, *η_p_^2^* = *0.917*, indicating that the new object was most frequently fixated at the first fixation. There was a main effect of mask condition, *F*(3, 33) = 33.651, *p*<.001, *η_p_^2^* = *0.754*, with a higher probability of fixating new objects in the no mask condition compared with the two other mask conditions and the baseline viewing rate. Most importantly, a visual mask had differential effects on the probability of fixating new objects across the four ordinal fixation positions, which was confirmed by a significant interaction between the ordinal fixation position and mask condition, *F*(9, 99) = 26.487, *p*<.001, *η_p_^2^* = *0.707*. More specifically, the pairwise comparison with Bonferroni correction showed that, at Fixation 1, participants fixated on the new object more frequently in the no mask condition relative to either the 50 ms or the 300 ms mask conditions, *p* = .005 and *p* = .012 respectively. In addition, the probabilities of fixating new objects at the no mask, 50 ms mask and 300 ms mask conditions were all significantly higher than the baseline viewing rate at Fixation 1, *p*<.001; *p* = .005; and *p*<.001 respectively. The results at Fixation 2 were contrary to the results at Fixation 1. Participants fixated new objects less often in the no mask condition than either the 50 ms (*p* = .031) or 300ms mask conditions (*p* = .025) at Fixation 2. Moreover, the probabilities of fixating new objects for the 50ms-mask and 300ms-mask conditions were significantly higher than the baseline viewing rate, *p* = .025, and *p*<.001 respectively; but the probability for the no mask condition was not different from the baseline viewing rate, *p*>.05. Results at Fixation 3 were similar to those at Fixation 2: the probabilities of fixating new objects in the 50ms-mask and 300ms-mask conditions were significantly higher than that of the no-mask condition (*p* = .004, and *p* = .002 respectively). However, none of them were different from the baseline viewing rate, all *p*s>.05. For Fixation 4, the probability of fixating new objects in the no-mask condition was lower than the baseline viewing rate, *p* = .049, but there were no other significant differences, all *p*s>.05.

Fore-mentioned results clearly show that, when there was a mask, the prioritization of the onset occurred at both Fixations 1 and 2, whereas onsets captured attention only at Fixation 1 when there was no mask. In addition, Table 2 lists the average latencies of fixating on a new object, and the average number of trials in which onsets were fixated at each combination of four ordinal fixations and three mask conditions. These results clearly show that latencies of fixating on a new object with visual masks at Fixation 1 are much longer than the latency of fixating on a new object with no mask at Fixation 1. Thus the prioritization of onset under the presence of a visual mask is more long-lived than the onset capture under absence of a visual mask. However, since the latencies at Fixations 3 and 4 were based on a very limited amount of trials, we suggest caution when referring to these latencies.

**Table 2:** The average latencies of fixation on a new object and average number of trials in which onsets were fixated in the non-onset target condition of Experiment 1.

	No mask	50 ms mask	300 ms mask	Baseline
**The average latencies of fixating at onsets (SD is listed in parenthesis)**				
1^st^ ordinal fixation	312 (51)	370 (24)	317 (50)	336 (44)
2^nd^ ordinal fixation	466 (93)	551 (66)	477 (57)	559 (55)
3^rd^ ordinal fixation	659 (177)	747 (77)	624 (77)	768 (80)
4^th^ ordinal fixation	987 (184)	970 (171)	1003 (277)	946 (83)
**The average number of trials in which onsets were fixated and baseline**				
1^st^ ordinal fixation	31.7 (10.4)	18.8 (8.7)	19.6 (5.5)	6.6 (1.5)
2^nd^ ordinal fixation	4.4 (2.3)	7 (3.9)	8.6 (2.9)	5.9 (1.9)
3^rd^ ordinal fixation	2.7 (1.6)	3.6 (2.3)	4.8 (2.6)	4.4 (1.9)
4^th^ ordinal fixation	1.5 (0.5)	1.6 (0.8)	2.1 (1.5)	2.2 (1.3)

We also analyzed the mean latency of the first fixation on a new object for correct trials. The mean latency of the first fixation on a new object was shorter when there was no mask (*M* = 370 ms after onset) relative to when there was a 50 ms mask (*M* = 484 ms after onset) or a 300 ms mask (*M* = 445 ms after onset), *F*(2, 22) = 17.425, *p*<.001, *η_p_^2^* = *0.613*. Therefore, the visual mask delays the onset capture because it increases the latency of fixating on the new object. A further pairwise comparison with Bonferroni correction showed the latency was shorter when there was no mask compared to both a 50 ms mask and a 300 ms mask (all *ps* = .001), but there was no significant difference between the 50 ms and 300 ms masks (*p* = .315). These results indicate that visual masks delayed the onset capture.

We also analyzed the *number of fixations to first look at a new object* because this number of fixations intervening between the onset of the new object and an observer's first fixation on that object is another measure of how quickly the onset is prioritized [30], [31]. On average, the new object was first viewed sooner if there is no mask (*M* = 1.3 fixations after onset) than if it appeared after the 50 ms mask (*M* = 1.6 fixations after onset) or after the 300 ms mask (*M* = 1.7 fixations after onset), *F*(2, 22) = 9.641, *p*<.001, *η_p_^2^* = *0.467*.

Figure 3B illustrates the probability that the new object was first fixated at each of the four ordinal fixation positions, given that it was fixated at all, broken down by the three mask conditions. The data illustrated in Figure 3B is different from that in Figure 3A in two aspects. First, Figure 3B includes only trials in which onsets were fixated, whereas all trials are included in Figure 3A. For example, all 64 trials with no visual mask in the non-onset target condition were used as denominator to compute probability in Figure 3A. However, only about 42 trials (65.9%) are included as denominator in Figure 3B because onsets were fixated on average for 42 trials when there was no mask in the non-onset target condition. Second, a re-fixation on the new object was considered as a second fixation on the new object. Thus these re-fixations were excluded from analyses in Figure 3B. In contrast, a re-fixation on the new object was counted as a new fixation on the new object and included in Figure 3A.

Thus Figure 3B illustrates how the probability distribution of first look to the new object over the four ordinal fixation positions differs across the three mask conditions. The probability distribution is another measure of how quickly the onset is prioritized. Note that in the no mask condition, 98.6% of all first looks to the new object occurred in the first four fixations after its appearance. In the 50 ms mask condition, this rate was 98.7%. In the 300 ms mask condition, this rate was 97.3%. The overall probabilities that the first look to the new object over the four ordinal fixation positions were the same for the three mask conditions, *F*(2, 22)<1, *p* = .483, *η_p_^2^* = *0.064*. As Figure 3B clearly shows, the first look to the new object occurred mostly at the first fixation after onset and that probability decreased as the ordinal fixation position increased, *F*(3, 33) = 140.37, *p*<0.001, *η_p_^2^* = *0.927*. Most importantly, there was an interaction between the ordinal fixation position and the mask condition, indicating that these probabilities of first look at new objects across ordinal fixations did not descend equally under the three mask conditions, *F*(6, 66) = 12.289, *p*<0.001, *η_p_^2^* = *0.528*. More specifically, at Fixation 1, the mean probability of the first fixation to onset was 61.8% in the 50 ms mask condition and 57.5% in the 300 ms mask condition, which were significantly lower than that in the no mask condition (the probability was 81.3%), confirmed by a pairwaise comparison with Bonferroni correction, *p* = 0.011 and *p* = 0.001 respectively. In contrast, at Fixation 2, the probability of first fixation on onset was enhanced by either 50 ms (*M* = 22.8%) or 300 ms masks (*M* = 24%) relative to no mask (*M* = 12.2%), *p* = 0.052 and *p* = 0.003 respectively. The same pattern also occurred at Fixation 3, the probability of first fixation to onset in the 50 ms mask condition (*M* = 11%) or the 300 ms mask condition (*M* = 11.9%) was higher than that in the no mask condition (*M* = 3.5%), *p* = 0.02 and *p*<0.001 respectively. At Fixation 4, the probability of first fixation to onset in the 50 ms mask condition (*M* = 3.1%) and the 300 ms mask condition (*M* = 3.9%) were not different from that in the no mask condition (*M* = 1.7%), all *p*s>0.05. In conclusion, it took participants more saccades and longer time to first fixate on a new object when there was a mask relative to when there was no mask, indicating a slower prioritization of new objects with the presence of masks.

#### Oculomotor capture in the onset target condition

**Figure 4:**
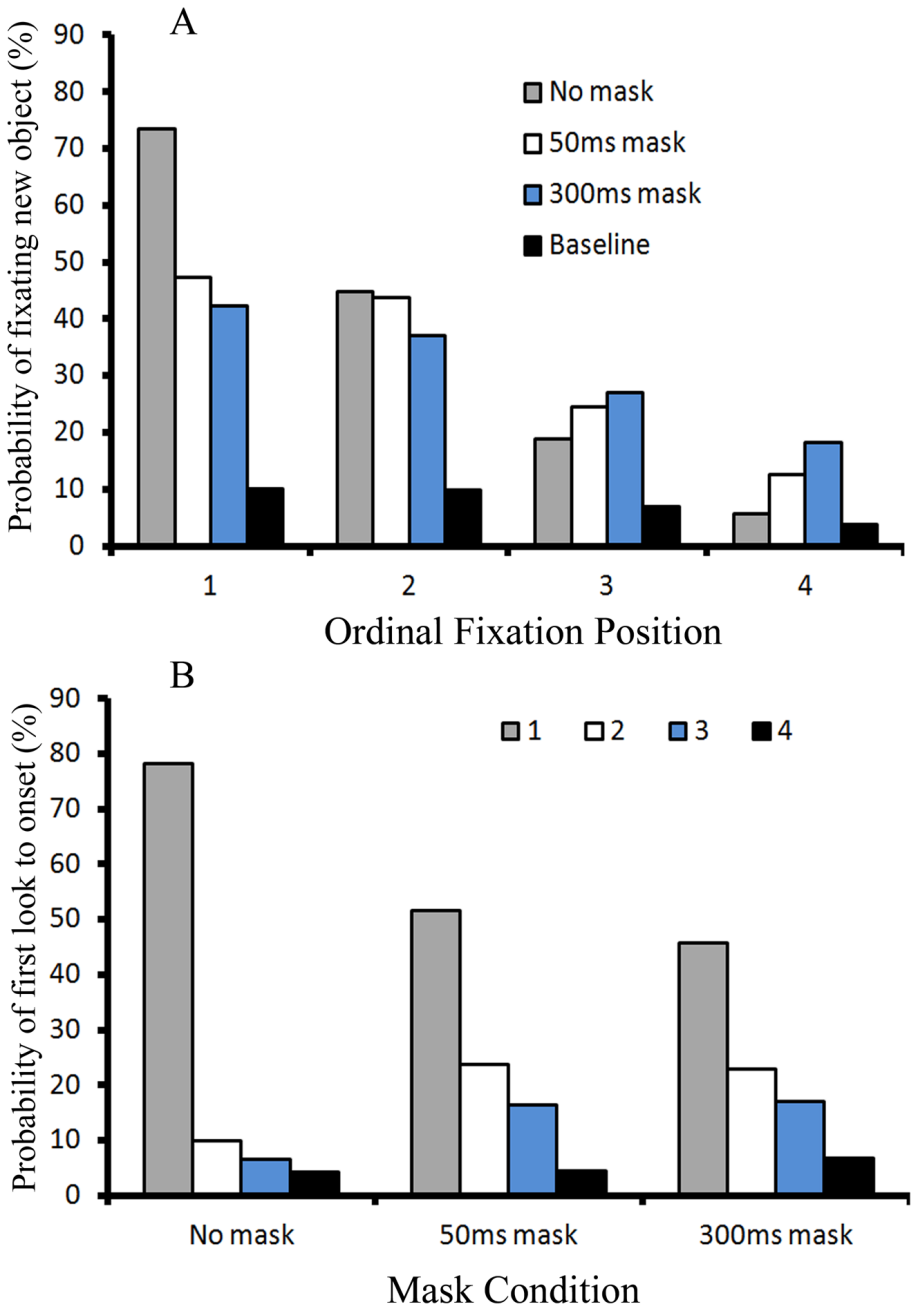
Fixation results under the onset target condition of Experiment 1. Panel A illustrates the probability of fixating new objects under the onset target condition in Experiment 1; Panel B illustrates the probability of first look to onset for the three mask types under the onset target condition.

When the new object was a target (the onset target condition), the probability of fixating new objects is illustrated as a function of the ordinal fixation position and mask condition in Figure 4A. A 4 (ordinal fixation position) X 4 (three mask conditions plus the baseline viewing rate) ANOVA revealed a main effect of ordinal fixation position, *F*(3, 33) = 37.997, *p*<.001, *η_p_^2^* = *0.775*, indicating that the onset target was most frequently fixated at the first fixation. There was a main effect of mask condition, *F*(3, 33) = 88.623, *p*<.001, *η_p_^2^* = *0. 89*, with a higher probability of fixating new objects in all three mask conditions than the baseline viewing rate. But visual masks had differential effects on the probability of fixating onset target across the four ordinal fixation positions, which was confirmed by a significant interaction between the ordinal fixation position and mask condition, *F*(9, 99) = 10.009, *p*<.001, *η_p_^2^* = *0.476*. More specifically, the pairwise comparisons with Bonferroni correction showed that, at Fixation 1, participants fixated on the onset target more frequently in the no mask condition relative to either the 50 ms or the 300 ms mask conditions, *p* = .042, and *p* = .008, respectively. Contrary to the results at Fixation 1, participants fixated onset target equally often for all three mask conditions at the other fixations (Fixations 2–4), all *p*s>.05. When compared with the baseline viewing rate, the probabilities of fixating on onset targets at the four ordinal fixations for the 300ms-mask condition were significantly higher than the baseline viewing rate, *all ps*<.05. For the 50ms-mask condition, the probabilities of fixating on onset targets at each of the first three ordinal fixations were significantly higher than the baseline viewing rate, *all ps*<.01. For the no-mask condition, the probabilities of fixating on onset targets at each of the first two ordinal fixations were significantly higher than the baseline viewing rate, *all ps*<.005. Though onset capture was self evident in all three mask conditions, the prioritization of the onset target is not due to onsets per se. Instead these high probabilities of fixating on the onset target were partly due to the fact that new objects were targets.

Table 3 lists the average latencies of fixation on a new object, and the average number of trials in which onsets were fixated at each combination of four ordinal fixations and three mask conditions in the onset target condition of Experiment 1.

**Table 3:** The average latencies of fixation on a new object and average number of trials in which onsets were fixated in the onset target condition of Experiment 1.

	No mask	50 ms mask	300 ms mask	Baseline
**The average latencies of fixating at onsets (SD is listed in parenthesis)**				
1^st^ ordinal fixation	323 (56)	380 (76)	339 (58)	336 (64)
2^nd^ ordinal fixation	500 (128)	545 (68)	489 (42)	582 (96)
3^rd^ ordinal fixation	643 (137)	769 (89)	731 (193)	762 (139)
4^th^ ordinal fixation	972 (134)	1014 (225)	827 (134)	969 (229)
**The average number of trials in which onsets were fixated and baseline**				
1^st^ ordinal fixation	11.6 (3.7)	7.5 (3.3)	6.8 (3.0)	1.5 (0.4)
2^nd^ ordinal fixation	1.9 (1.2)	3.6 (1.3)	3.4 (1.3)	1.0 (0.3)
3^rd^ ordinal fixation	1.6 (0.8)	2.6 (2.0)	2.8 (1.9)	0.5 (0.3)
4^th^ ordinal fixation	1.2 (0.4)	1.6 (0.5)	1.3 (0.7)	0.3 (0.2)

Again, the mean latency of the first fixation on the onset target for correct trials was shorter when there was no mask (M = 398 ms after onset) relative to when there was a 50 ms mask (M = 558 ms after onset) or a 300 ms mask (M = 547 ms after onset), *F*(2, 22) = 22.535, *p*<.001, *η_p_^2^* = *0.*672. Further pairwise comparisons with Bonferroni correction showed the latency was shorter when there was no mask compared to when there was either a 50 ms mask or a 300 m mask (all *ps*<.005), but there was no significant difference between the 50 ms and 300 ms mask conditions (p = 1).

On average, the onset target was first viewed sooner if there was no mask (*M* = 1.4 fixations after onset) than if it appeared after the 50 ms mask (*M* = 1.9 fixations after onset) or after the 300 ms mask (*M* = 2.2 fixations after onset), *F*(2, 22) = 11.286, *p*<.001, *η_p_^2^* = *0.506*.

Figure 4B illustrates the probability that the onset target was first fixated at each of the first four ordinal fixation positions, given that it was fixated at all, broken down by the three mask conditions. Note that in the no mask condition, 98.9% of all first looks to the new object occurred in the first four fixations after its appearance. In the 50 ms mask condition, this rate was 96.3%. In the 300 ms mask condition, this rate was 92.5%. The overall probability that the first look was to the new object was highest for the no-mask condition, *F*(2, 22) = 4.187, *p* = .029, *η_p_^2^* = *0.276*. The first look to the new object occurred mostly at the first fixation after onset and that probability decreased as the ordinal fixation position increased, *F*(3, 33) = 67.74, *p*<0.001, *η_p_^2^* = *0.86*. Most importantly, the probability of first look to onset target decreased unevenly across the first four ordinal fixation positions for the three mask conditions, *F*(6, 66) = 7.995, *p*<0.001, *η_p_^2^* = *0.421*. The overall pattern is same as that of the probability of first look to new objects in the no onset target condition. At the first fixation, the mean probability of first fixation to the onset target in the 50 ms mask (*M* = 51.6%) and the 300 ms mask conditions (*M* = 45.8%) was lower than that of the no mask condition (the probability was 78.2%), confirmed by a pairwaise comparison with Bonferroni correction, *p* = 0.043 and *p* = 0.011 respectively. At Fixation 3, the probability of first fixation on the onset target in the 300 ms mask condition (*M* = 17%) was significantly higher than that in the no mask conditions (*M* = 6.5%), *p*  = 0.036. There were no other significant differences between the three mask conditions across Fixations 2–4, all *p*s>0.05.

#### Comparison between the non-onset target and onset target conditions

For simplicity, we performed a 2 (non-onset target vs. onset target) x 3 (three mask conditions) ANOVA for the probability of fixating onsets at the first ordinal Fixation in Experiment 1. This analysis actually reveals the effect of task relevancy upon onset capture rather than the onset capture effect. The ANOVA showed a significant effect of the target onset conditions, *F*(1,11) = 32.089, *p*<.001, *η_p_^2^* = .745; and a significant main effect of the mask conditions, *F*(2,22) = 16.594, *p*<.001, *η_p_^2^* = .601. The interaction was significant too, *F*(2,22) = 3.551, *p* = .046, *η_p_^2^* = .244. More specifically, at Fixation 1, participants fixated an onset more often in the onset target condition than in the non-onset target condition with no mask (*p*<.001), with a 50 ms mask (*p* = .001) and with a 300 ms mask (*p* = .023). These results indicate that the task relevancy of onsets (whether an onset is target or not) modulates the probability of fixating on onsets.

### Discussion

The present experiment showed that luminance transient associated with onsets is important for capturing attention because the visual mask significantly reduced the probability of fixating new objects at the first ordinal fixation position if compared with that under the no mask condition. However, in sharp contrast to the luminance transient theory, participants still fixated more often on the new objects than chance level even with the presence of a visual mask. This indicates that an onset still has attentional priority even when a visual mask disrupts the unique luminance transient associated with onset. Thus it is consistent with the dual processes theory in the sense that the prioritization of onset is not solely relying on the unique luminance transients associated with onset. In addition, the present experiment showed that, when there was a mask, the prioritization of the onset occurred at Fixations 1 and 2, whereas onsets captured attention only at the Fixation 1 when there was no mask. Thus the prioritization of onset under the presence of a visual mask is more long-lived than the short-lived onset capture under absence of a visual mask. As the dual processes theory would predict, the prioritization of onset with the presence of a visual mask is more sluggish and long-lived than the rapid and short-lived onset capture when a mask is absent.

## Experiment 2

Experiment 1 demonstrated that a new object still has an advantage compared with old objects even when the unique luminance transients associated with onsets are disrupted by visual masks. These results are consistent with the dual processes model of onset capture which proposes that oculomotor capture of onset is a joint effect of the bottom-up capture driven by luminance transients and the top-down prioritization based on memory. Visual masks are only expected to disrupt the bottom-up capture of attention driven by luminance transients. Thus, visual masks can only reduce but not completely wipe out the onset advantage.

Consistent with previous studies [30], [31], Experiment 1 showed that the prioritization of new objects under disruption of visual masks was more sluggish and prolonged than when there was no visual mask. This finding indicates that the memory-based prioritization of new objects is slower and more long-lasting than involuntary capture driven by luminance transients. In addition, Brockmole et al. (2008) demonstrated that the memory-based prioritization is influenced by object identity and the viewing time of a scene [32]. Their results provide indirect evidence that the memory-based prioritization is under top-down control. However, it is still unknown whether this memory-based prioritization of onset is truly under top-down guidance of target location. To examine this possibility, we set the probability with which a target appears as a new object to be zero in Experiment 2. Since the new object was never a target here, the probability of fixating new objects under the no mask condition should exceed the baseline viewing rate after onset, mainly reflecting a bottom-up capture of attention [34], [35]. However, the probability of fixating the new object with the presence of a mask should be as low as the baseline viewing rate if the memory-based prioritization of onset is under top-down control.

### Methods

#### Participants

Sixteen new undergraduate and graduate students (6 males, 23.56±2.42 yrs) participated in this experiment. All of them had normal or corrected-to-normal vision. Participants were naïve with regard to the purpose of the experiment. Each participant received cash compensation for their participation.

#### Apparatus, procedure and design

Experiment 2 was identical to Experiment 1, with one exception. The target letter was presented at any of four locations where placeholders appeared. Thus the target letter was always a non-onset letter, and the new object was always an irrelevant letter. Participants were told that the target letter always appeared at one of four cued locations and was never a new object. As a result, the experiment has only one within-subject variable: the mask condition (no mask, presence of a 50 ms mask, or presence of a 300 ms mask). Sixty-four trials were assigned to each of the three mask conditions.

Participants served in one 25 minute session divided into four blocks. The first block consisted of 32 trials for participants to practice, and each of the other three blocks consisted of 64 trials, for a total of 192 trials.

### Behavioral Results

**Figure 5:**
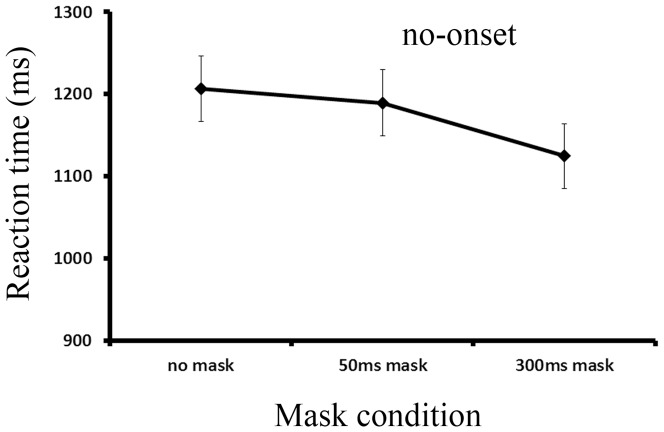
The mean reaction time of all conditions in Experiment 2.

The mean RTs of Experiment 2 are illustrated as a function of the mask condition in Figure 5. An ANOVA revealed a significant effect of mask condition, F (2, 30) = 8.146, *p* = .001, *η_p_^2^* = *0.352*, with a slower response to targets in the no mask condition than in the 300 ms mask condition. These results indicate that a visual mask probably reduced the attentional priority of onsets, resulting in less onset interference with the search for the non-onset target. Alternatively, faster RTs to the non-onset target in the 300 ms mask condition could also reflect the effect of a temporal warning signal of masks. A 300 ms mask provides a longer period for preparation for the targets than with a 50 ms mask or without masks at all.

The mean accuracy of Experiment 2 is listed in Table 4. The average accuracies in all conditions were above 97%. The ANOVA test revealed no effects of mask duration, F (2, 30) = 0.413, *p* = .665, *η_p_^2^* = 0.027.

**Table 4:** The mean accuracy of the three mask conditions in Experiment 2.

	No mask	50 ms mask	300 ms mask
**No-onset**	98.14±.52	97.85±.53	98.14±.41

### Eye Movements Results

Analysis of eye movements focused on the probability of fixating the new object region of the scene. Four ordinal fixations following the appearance of the new object (denoted Fixations 1, 2, 3 and 4) were examined. We also used the probability that participants fixated on a non-onset and non-target letter as the *baseline viewing rate*.

#### Oculomotor capture

**Figure 6:**
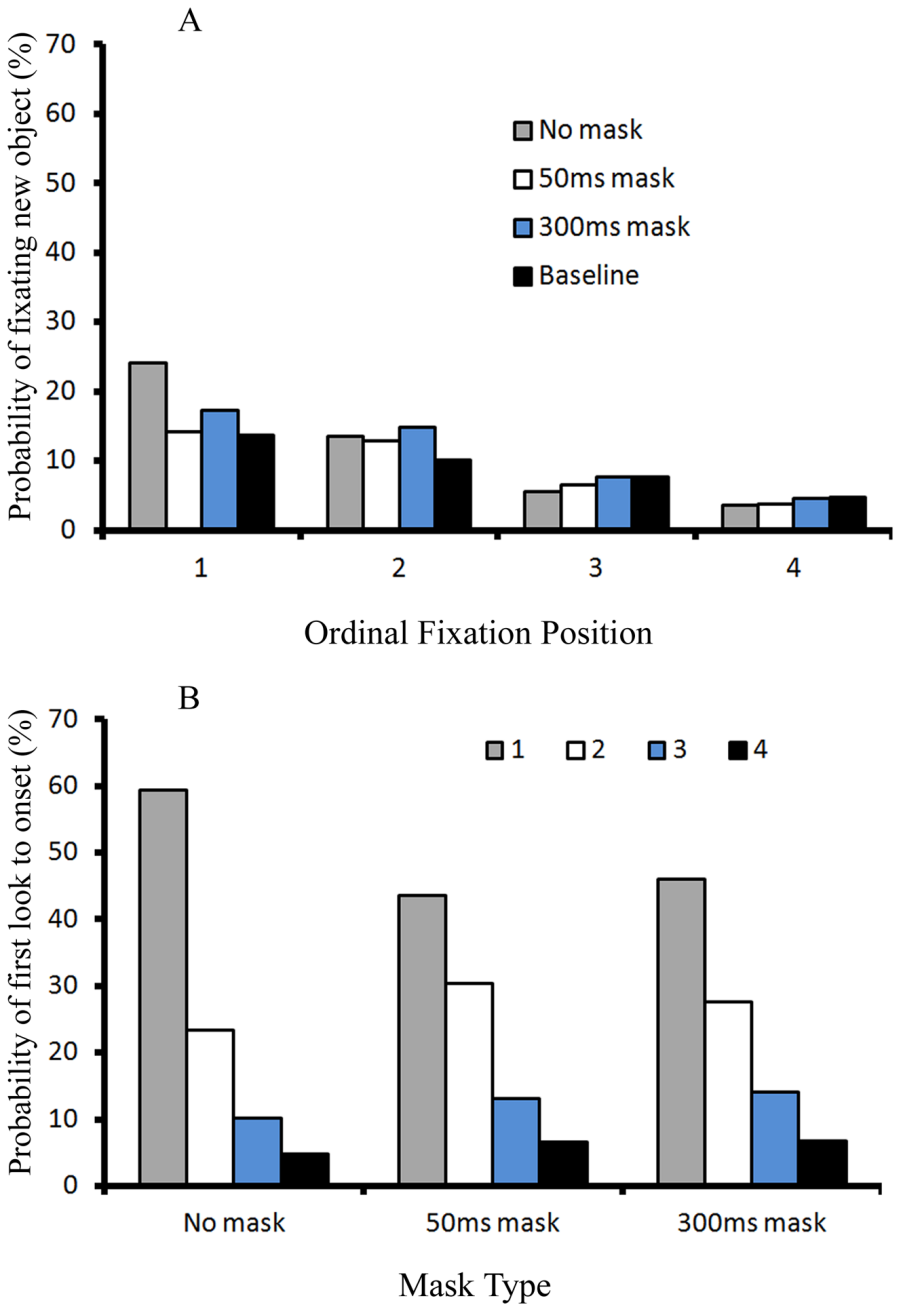
Fixation results of Experiment 2. Panel A illustrates the probability of fixating new objects in Experiment 2; Panel B illustrates the probability of first look to onset for the three mask types in Experiment 2.

The probability of fixating new objects is illustrated as a function of the ordinal fixation position and mask condition in Figure 6A. A 4 (ordinal fixation position) X 4 (three mask conditions plus the baseline viewing rate) ANOVA revealed a significant main effect of ordinal fixation position, *F*(3, 45) = 48.958, *p*<.001, *η_p_^2^* = *0.765*, indicating that the new object was most frequently fixated at the first fixation. There was a main effect of mask condition, *F*(3, 45) = 4.697, *p* = .006, *η_p_^2^* = *0. 238*. Most importantly, visual masks had differential effects on the probability of fixating new objects across the four ordinal fixation positions for the three mask conditions, which was confirmed by a significant interaction between the ordinal fixation position and mask condition, *F*(9, 135) = 5.604, *p*<.001, *η_p_^2^* = *0.272*. More specifically, a pairwise comparison with Bonferroni correction showed that, at Fixation 1, participants fixated on the new object more frequently in the no mask condition relative to the two other mask conditions, *p* = .006, and *p* = .072, respectively. At Fixation 1, only the probability of fixating new objects in the no-mask condition was significantly higher than the baseline viewing rate, *p* = .023; but the probability of fixating new objects in both the 50ms-mask and 300ms-mask conditions were not significantly different from the baseline viewing rate, all *ps*>.05. From Fixation 2 to Fixation 4, participants fixated on new objects almost equally often for the three mask conditions across these three ordinal fixations, all *ps*>.05. In addition, the probabilities of fixating new objects for the three mask conditions at each of the three ordinal fixations were not different from their corresponding baseline viewing rate, all *ps*>.05.

Table 5 lists the average latencies of fixation on a new object, and the average number of trials in which onsets were fixated at each combination of four ordinal fixations and three mask conditions in Experiment 2.

**Table 5:** The average latencies of fixation on a new object and average number of trials in which onsets were fixated for all conditions in Experiment 2.

	No mask	50 ms mask	300 ms mask	Baseline
**The average latencies of fixating at onsets (SD is listed in parenthesis)**				
1^st^ ordinal fixation	363 (98)	386 (100)	334 (102)	356 (85)
2^nd^ ordinal fixation	528 (154)	556 (104)	504 (85)	586 (97)
3^rd^ ordinal fixation	732 (255)	748 (165)	685 (136)	777 (113)
4^th^ ordinal fixation	994 (180)	1032 (213)	927 (164)	1010 (188)
**The average number of trials in which onsets were fixated**				
1^st^ ordinal fixation	15.1 (7.3)	8.9 (6.1)	10.8 (4.7)	8.4 (2.4)
2^nd^ ordinal fixation	7.6 (4.8)	7.1 (3.2)	7.1 (4.0)	4.8 (1.5)
3^rd^ ordinal fixation	2.7 (1.1)	3.5 (1.9)	3.6 (1.8)	3.6 (1.8)
4^th^ ordinal fixation	2.5 (1.3)	2.8 (2.0)	2.5 (1.6)	2.1 (1.1)

The mean latency of the first fixation on irrelevant new objects for correct trials was shorter when there was no mask (*M* = 488 ms after onset) or a 300 ms mask (*M* = 522 ms after onset) relative to when there was the 50 ms mask (*M* = 587 ms after onset), *F*(2, 30) = 10.564, *p*<.001, *η_p_^2^* = *0.413*. A further pairwise comparison with Bonferroni correction showed the latency was shorter when there was either no mask or a 300 m mask compared to when there was a 50 ms mask (*p* = .003 and *p* = .011 respectively), but there was no significant difference between the no mask condition and the 300 ms mask condition (*p* = .460). This pattern indicated that the 50 ms mask condition did slow down the prioritization of onsets compared with the no mask condition. However, the 300 ms mask probably provided a longer period for participants to prepare for the visual search task than the 50 mask. As a result, onsets were fixated faster when there was a 300 ms mask than when there was a 50 ms mask, though the probability of fixating on onsets are suppressed to the same extent in the two mask conditions.

We also analyzed the *number of fixations to first look at a new object.* On average, the new object was first viewed sooner if there was no mask (*M* = 1.69 fixations after onset) than if it appeared after the 50 ms mask (*M* = 2.05 fixations after onset) or after the 300 ms mask (*M* = 2.02 fixations after onset), *F*(2, 30) = 9.37, *p* = .001, *η_p_^2^* = *0.384*.

Figure 6B illustrates the probability that the new object was first fixated at each of the first four ordinal fixation positions, given that it was fixated at all, broken down by the three mask conditions. Note that in the no mask condition, 97.7% of all first looks to the new object occurred in the first four fixations after its appearance. In the 50 ms mask condition, this rate was 93.7%. In the 300 ms mask condition, this rate was 94.6%. The overall probabilities that the first look to the new object at the first four ordinal fixation positions were almost the same for the three mask conditions, *F*(2, 30) = 2.55, *p* = .095, *η_p_^2^* = *0.0145*. The first look to the new object occurred mostly at the first fixation after onset and that probability decreased as the ordinal fixation position increased, *F*(3, 45) = 41.328, *p*<0.001, *η_p_^2^* = *0.734*. Most importantly, there was an interaction between ordinal fixation position and mask condition, *F*(6, 90) = 4.145, *p* = 0.001, *η_p_^2^* = *0.217*, indicating that the probability of first look to onset target decreased unevenly across the first four ordinal fixation positions for the three mask conditions. More specifically, at Fixation 1, the probabilities of first fixating at onset in both the 50 ms and the 300 ms mask conditions (mean probability of first fixation to onset was 43.6% in the 50 ms mask condition and 46% in the 300 ms mask condition) were reduced compared with that in the no mask condition (the probability was 59.3%), confirmed by a pairwise comparison with Bonferroni correction, *p* = 0.024 and *p* = 0.019 respectively. But at the other three ordinal fixations, the probabilities of first fixation to onset in the three mask conditions were not significantly different from each other, all *p*s>0.05. This pattern indicated that a visual mask still makes participants slower to fixate on the onset even though they do not fixate on the onset more often than the baseline viewing rate when there is a mask.

### Discussion

An onset still captured attention involuntarily when there was no visual mask in between cue and target. This indicated that luminance-driven capture can resist top-down control and is involuntary. More importantly, with the presence of a visual mask, the memory-based prioritization of onset was eliminated when participants were aware that onset was never a target.

## Comparison between Experiments 1 and 2

In order to compare oculomotor capture by onsets between Experiments 1 and 2, a 2 (Fixations 1 and 2) X 4 (three mask conditions plus the baseline viewing rate) X 2 (two experiments) ANOVA was performed. The between experiments main effect (between Experiments 1 and 2) showed that a new object in Experiment 1 was more likely to capture attention than that in Experiment 2; *F*(1, 26) = 13.217, *p* = .001, *η_p_^2^* = 0.337, indicating a higher overall rate of onset capture when the target was an onset letter. Most importantly, there was a three way interaction, F(3, 78) = 12.919, *p*<.001, *η_p_^2^* = 0.332, indicating that the probabilities of fixating new objects at two ordinal fixation positions for three mask conditions differ in the two experiments. Specifically, at Fixation 1, participants fixated on an irrelevant onset more often in the no mask condition of Experiment 1 than in the same condition of Experiment 2 (*p*<.001), also for the 50 ms mask (*p* = .001) and 300 ms mask (*p*<.001) conditions. In contrast, the baseline viewing rate in Experiment 1 was lower than that in Experiment 2 (*p* = .025). These results indicate that onset advantage was reduced when observers were aware that onsets were completely irrelevant. However, at Fixation 2, the probabilities of fixating new objects were not different between the experiments for all three mask conditions and baseline, all *p*>.220.

In addition, a comparison between experiments showed that it took more saccades for participants to fixate on an irrelevant onset in Experiment 2 (*M* = 1.92 fixations after onset) than a relevant onset in Experiment 1 (*M* = 1.54 fixations after onset), *t*(26) = 3.058, *p* = .005. They were also slower to fixate on an irrelevant onset in Experiment 2 (*M* = 532 ms after onset) than a relevant onset in Experiment 1 (*M* = 433 ms after onset), *t*(26) = 3.472, *p* = .002. This indicates that onset advantage was modulated by top-down control.

## General Discussion

Experiment 1 showed that participants tended to fixate more frequently on new objects than chance when there was no visual mask, replicating the classical finding of onset capture. Partially consistent with previous findings [14], [15], Experiment 1 showed that luminance transient associated with onsets did play an important role in capturing attention because the visual mask significantly reduced the probability of fixating on new objects at the first ordinal fixation position compared with that under the no mask condition. However, contrary to the luminance transient account, even with the presence of a visual mask between the cue and target (there was no unique luminance transient associated with onset), participants still fixated more often on the new object than chance level in Experiment 1. This finding along with a faster response to onset targets relative to non-onset targets with the presence of visual mask indicates that the prioritization of onset is not solely relying on the unique luminance transient associated with the onset. The present results are better explained by the new object theory of onset capture [16]–[18], [20] rather than the luminance transient account [14], [15]. In addition, Experiment 1 showed that the prioritization of onset occurred at both the first and second ordinal fixations with the presence of visual masks, whereas onsets only captured attention at the first ordinal fixations when there was no mask. Thus the prioritization of onset under the presence of a visual mask is more sluggish and long-lived than the rapid and short-lived onset capture under absence of a visual mask. This finding is also consistent with the previous findings that reported even more sustained prioritization of new objects in a task of viewing a natural scene [13], [30]–[32]. Thus, even with a visual search task, Experiment 1 confirmed two temporal characteristics of the memory-based prioritization of onset relative to the onset capture mainly driven by luminance transients: slower and longer.

Though the onset still captured attention involuntarily when there was no visual mask, a visual mask did completely eliminate the prioritization of onset when participants were aware that the onset was never a target in Experiment 2. This finding along with results of Experiment 1 indicates that there were truly two distinctive processes of onset capture regarding its intentionality. The transient-driven capture of onset is involuntary because it occurred even when participants were aware that the onset is completely irrelevant to the task in Experiment 2. In contrast to the involuntary capture mainly driven by unique luminance transient, the memory-based prioritization of onset is truly under top-down control because its occurrence is contingent upon the task relevancy of the onset. More specifically, the memory-based prioritization of onset occurred under both mask conditions when an onset letter could be a target equally often as any non-onset letters in Experiment 1. However, this prioritization of onset was absent from both mask conditions when an onset letter was never a target in Experiment 2. This finding that memory-based prioritization of onset is voluntarily controlled is also consistent with previous findings that cognitive load modulates the onset capture [8], [36], [37].

As we pointed out in the introduction, the present study is different from the Brockmole et al studies [30], [31]. Thus we found three important new findings. First, Brockmole and colleagues used saccades to suppress global luminance transient signals, whereas visual masks in the present study only make the luminance transient associated with the new object less distinguishable from the transients associated with pre-existing objects. The present findings indicate that visual masks are effective in suppressing onset capture by disrupting the unique luminance transient associated with onsets. Alternatively, the visual mask could have served as a temporal warning signal and made participants better prepared for the upcoming target display. This hypothesis can explain why participants responded faster to the non-onset target when there was a mask compared to when there was no mask. It can explain why onsets were fixated faster when there was a 300 ms mask than when there was a 50 ms mask in Experiment 2. However, the temporal warning signal hypothesis cannot explain why the RTs of onset targets significantly increase with the presence of a visual mask. Neither could it explain why the latency of first fixation on onsets with no mask is faster than that with a 50 ms mask and a 300 ms mask in the non-onset target condition. Therefore, a visual mask mainly suppresses onset capture rather than makes participants better prepared in the present study. Admittedly, it is also possible that a 300 ms mask provides more time for the memory-based prioritization of onset to initiate than a 50 ms mask. As a result, onsets were fixated faster with a 300 ms mask than with a 50 ms mask.

Secondly, unlike the Brockmole et al. studies, the present study directly manipulated the probability that onset can be target. This helped to reveal the extent to which dual processes of onset capture are modulated by the top-down control setting based on the knowledge of target location. The attentional capture driven by the unique luminance transients occurred when participants were aware that the onset is never a target in Experiment 2, and was thus involuntary. However, the memory-based prioritization of onset is truly under top-down control because its occurrence is contingent upon the task relevancy of onset. Finally, the present study used a visual search task instead of a natural scene viewing task. It is evident that participants were much less likely to re-fixate an object in a visual search task than in a scene viewing task. However, even with a visual search task, we confirmed the distinctive temporal characteristics for dual processes of onset capture. The involuntary capture guided by luminance transient is rapid and short-lived, while the memory-based voluntary prioritization is relatively sluggish and long-lived. The present study and some previous studies [30], [31] reach a similar conclusion by using distinctive approaches, thus providing converging evidence for the dual processes model of onset capture.

The present study might help reconcile two long-lasting debates on onset capture. First, researchers have been debating whether onset capture is solely caused by the unique luminance transients of onset [14], [15] or the appearance of a new perceptual object [16], [17], [20]. The present results show that both causes are critical for producing onset capture. The other debate concerns whether onset capture is involuntary [1], [3], [7] or somehow under voluntary control [21]–[23]. The present results indicate that onset capture is due to a joint effect of two distinctive processes. One is a rapid involuntary capture driven by unique luminance transients. This process dominates the onset capture when an onset is accompanied by a unique luminance transient. However, the transient-driven capture is also susceptible to top-down modulation because the transient-driven capture is suppressed in Experiment 2 relative to Experiment 1. The other process is a sluggish voluntary prioritization for a new object which relies on the memory representation of pre-existing objects. This memory-based prioritization of new objects grants attentional priority to onsets when the unique luminance transient of onset is either suppressed by saccades [30], [31] or eliminated by a visual mask [14], [16]. As a result, onset capture possesses two sets of seemingly contradicting characteristics: it can either be a rapid, short-lived and involuntary capture or a sluggish, long-lived and voluntary prioritization.

In summary, the present results are consistent with the dual processes theory of onset capture. In addition, the transient-driven capture of onset is involuntary, rapid and short-lived; whereas the memory-based prioritization of onset is voluntary, sluggish and relatively long-lived. However, it is still possible that other factors might contribute to onset capture. More research is needed in future.
